# Respiratory Cilia as a Therapeutic Target of Phosphodiesterase Inhibitors

**DOI:** 10.3389/fphar.2020.00609

**Published:** 2020-05-06

**Authors:** Marta Joskova, Juraj Mokry, Sona Franova

**Affiliations:** Department of Pharmacology, Jessenius Faculty of Medicine in Martin, Comenius University in Bratislava, Martin, Slovakia

**Keywords:** ciliary beat frequency, mucociliary clearance, calcium, nucleotides, phosphodiesterase, phosphodiesterase inhibitors, inhaled drug delivery

## Abstract

Mucociliary clearance is an essential airway defense mechanism dependent predominantly on the proper ciliary function and mucus rheology. The crucial role of cilia is evident in `a variety of respiratory diseases, as the ciliary dysfunction is associated with a progressive decline in lung function over time. The activity of cilia is under supervision of multiple physiological regulators, including second messengers. Their role is to enable a movement in coordinated metachronal waves at certain beat frequency. Ciliary function can be modulated by various stimuli, including agents from the group of beta_2_ agonists, cholinergic drugs, and adenosine triphosphate (ATP). They trigger cilia to move faster in response to elevated cytoplasmic Ca^2+^ originated from intracellular sources or replenished from extracellular space. Well-known cilia-stimulatory effect of Ca^2+^ ions can be abolished or even reversed by modulating the phosphodiesterase (PDE)-mediated breakdown of cyclic adenosine monophosphate (cAMP) since the overall change in ciliary beating has been dependent on the balance between Ca^2+^ ions and cAMP. Moreover, in chronic respiratory diseases, high ATP levels may contribute to cAMP hydrolysis and thus to a decrease in the ciliary beat frequency (CBF). The role of PDE inhibitors in airway cilia-driven transport may help in prevention of progressive loss of pulmonary function often observed despite current therapy. Furthermore, administration of selective PDE inhibitors by inhalation lowers the risk of their systemic effects. Based on this review we may conclude that selective (PDE1, PDE4) or dual PDE inhibitors (PDE3/4) increase the intracellular level of cyclic nucleotides in airway epithelial cells and thus may be an important target in the development of new inhaled mucokinetic agents. Further research is required to provide evidence of their effectiveness and feasibility regarding their cilia-modulating properties.

## Introduction

During last decades, a cilium has emerged as a key player in numerous physiological and developmental processes. Ciliary dysfunction results in a broad range of clinical diseases limiting the quality of patient´s life even despite an appropriate therapy. Therefore, cilia have become an important focus of interest in respirology. The exact mechanisms responsible for their motility and regulation have been elucidated recently, and the involvement of cyclic nucleotide (cAMP, cGMP) signaling in amplifying the Ca^2+^-dependent movement of motile cilia has been confirmed as one of the most important (Zagoory et al., 2002; Kogiso et al., 2017). This explains why inhibition of phosphodiesterases (PDEs), enzymes responsible for degradation of cAMP and cGMP, represents one of the targets for pharmacological modulation (Mokry et al., 2008) and improvement of ciliary dysfunction.

## Cilia

Cilia are microtubule-based organelles that have diverse motility and sensory functions. Although nearly all of cilia possess sensing role and transmit extracellular signals into intracellular biochemical responses, only some of them are able to move.

Motile cilia perform rotary motion or create wave-like planar beating based on the presence or absence of a central pair of microtubules surrounded by nine microtubule doublets of axoneme. Rotary motion additionally requires the absence of radial spokes (Shinohara et al., 2015). Adenosine triphosphate (ATP) as a source of energy is essential for the ciliary motion during which dynein motor proteins generate sliding movement between adjacent microtubules. Motile cilia were primarily found on ciliated epithelium lining the respiratory tract, fallopian tubes, brain ventricles, and the spinal cord. Except of their involvement in motility, all motile cilia play also sensory functions (Andrade et al., 2005; Teilmann et al., 2005; Lorenzo et al., 2008; Wodarczyk et al., 2009; Shah et al., 2009).

Immotile cilia (known also as “primary cilia”) act as sensory organelles of the cell, and their crucial role can be observed in various ciliopathies including retinal degeneration, anosmia, deafness, hepatobiliary, pancreatic cyst, and many others (Hou et al., 2002; Ward et al., 2003; Kulaga et al., 2004; Cano et al., 2006; Murga-Zamalloa et al., 2010; Grati et al., 2015).

**Figure 1:**
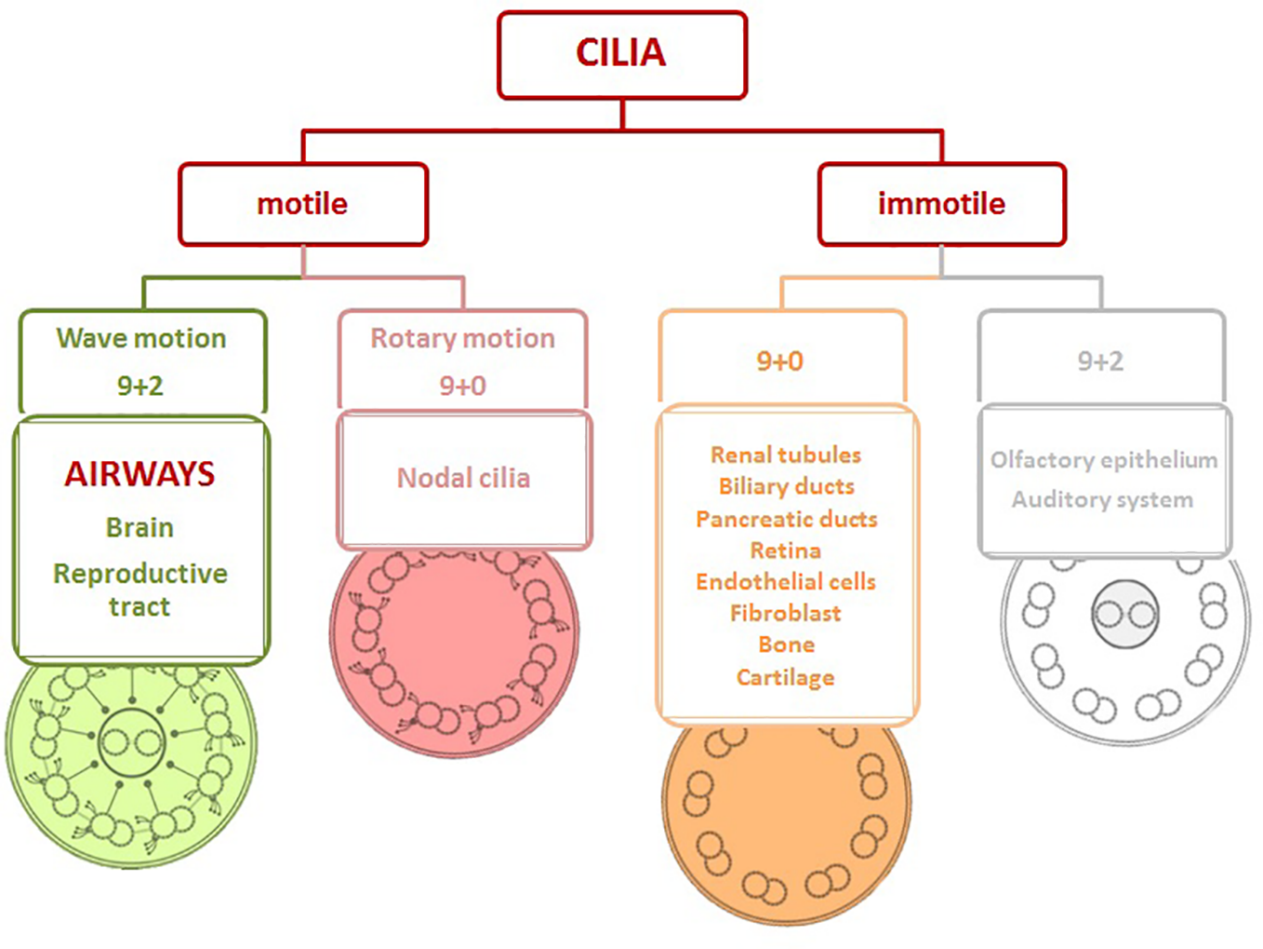
Structure of cilia viewed in cross-section and cilia distribution based on the motility in the body. Motile cilia with/without a central pair of microtubules (9 + 2/9+0), immotile cilia with/without a central pair of microtubules (9 + 2/9+0).

Cilia can be classified into four basic types (**Figure 1**): motile cilia with a central pair of microtubules (9 + 2), motile cilia without a central pair of microtubules (9 + 0), non-motile cilia with a central pair of microtubules (9 + 2), and non-motile cilia with absent central pair of microtubules (9 + 0) (Falk et al., 2015).

### Structure of Cilia

Motile cilia of airway epithelial cells contain a central pair of single microtubules surrounded by nine peripheral microtubule pairs (9 + 2) and protein complexes necessary for ciliary beating, such as axonemal dynein arms and radial spokes (Verhey et al., 2011). The dynein arms are generally classified into two distinct groups according to the number of heavy-chain motor units. The first group with the inner dynein arms is often situated along the axonemal lengths, although several “minor dyneins” were found near the ciliary base probably due to their role in bend initiation. The second group of the outer dynein arms is required to restart backward motion of cilium and change the ciliary beat pattern associated with increase of calcium levels in response to different signals (King, 2016). So, the change in the ciliary beat frequency (CBF) is dependent on the outer dyneins.

Normal ciliary ultrastructure can be visualized using a transmission electron microscopy (TEM). High-speed digital video analysis has improved measurement and quantification of CBF, while improvements in air–liquid interface culturing techniques have led to development of *in vitro* models to investigate mucociliary clearance.

### Ciliar Motility

The cilia of the airways beat in a highly coordinated and synchronized fashion across multiple ciliated cells. At the basal conditions the low CBF is dependent on the dynein ATPase activity of the axoneme with ability of cilia to increase it in the response to various stimuli (Ma et al., 2002). Calcium (Ca^2+^)–calmodulin complex could be considered as the key regulator of CBF linked with both nucleotides, cAMP (cyclic adenosine monophosphate) and cGMP (cyclic guanosine monophosphate), in the process of ciliary stimulation, although cAMP can also play a role in Ca^2+^-independent manner (Zagoory et al., 2002). In this cross-talk the cyclic nucleotides are essential for Ca^2+^ to be effective since disruption of nitric oxide (NO)–cGMP–protein kinase (PK) G pathway at any of the steps in the presence of high Ca^2+^ concentration eliminates its action (Schmidt and Salathe, 2011). Ca^2+^ is generally released from intracellular sources by inositol-3-phosphate (IP_3_) following stimulation of certain membrane receptors (e.g., purinergic P2Y2, cholinergic M_1_ and M_3_) or is transported from extracellular space *via* ion channels that mediate influx of Ca^2+^ to the ciliary cells (Schmidt and Salathe, 2011).

Ciliary response to “second messengers” is usually biphasic. During the initial phase the rise in CBF mediated *via* muscarinic receptors is Ca^2+^–calmodulin-dependent and mainly regulated by PKG. The second phase of CBF enhancement is induced by acetylcholine (Ach) with a sustained moderately elevated CBF, requiring PKG activation. However, this phase is controlled predominantly by axonemal PKA in a Ca2+-independent manner (Sanderson and Dirksen, 1989; Lansley et al., 1992; Kultgen et al., 2002; Zagoory et al., 2002; Schmid et al., 2007). Most enzymes and precursors involved in the ciliary motility are located at the base of the ciliary axoneme close to their site of action targeting phosphorylation and efficient regulation of the ciliary beating (Stout et al., 2007).

CBF can be considered as one of the crucial factors determining the rate of mucociliary clearance in daily life since even small frequency reduction (beats/s) may have clinical significance when considering clearance of secretions over hours. Furthermore, despite the normal CBF, the efficacy of mucociliary clearance is dependent also on the proper ciliary beat pattern. This is well documented in patients with primary ciliary dyskinesia (PCD) (Jorissen et al., 2000).

### Cilia in Mucociliary Clearance

Mucociliary clearance belongs to the group of defense mechanisms in the airways. In pathological conditions associated with CBF slowing (e.g., respiratory infection), the cough and the other antibacterial defense mechanisms can temporarily substitute it (Feldman et al., 2002; Bailey et al., 2012). Therefore, drug combinations of cough suppressants and agents with negative effects on the ciliary beating in the airways could be considered as unsuitable with strong clinical significance, as they negatively influence also reserve defense mechanism. Similarly, less risk for exacerbations of chronic bronchitis or chronic obstructive pulmonary disease (COPD) has been recently confirmed in patients taking mucolytics probably due to reduced mucus viscosity making it easier to expectorate (Poole et al., 2019). Mucolytics provide also additional direct cilio-stimulatory and bronchodilator effects without impact on the cough sensitivity, anti-inflammatory (Pappova et al., 2017; Frañová et al., 2019), antioxidant (Miyake et al., 1999) or immunomodulatory properties, in addition to the ability to reduce bacterial adhesiveness (Braga et al., 1999, Pappová et al., 2018).

The overall effect of mucociliary clearance is dependent on the proper ciliary function determined by the ciliary beat frequency, on the ciliary pattern, and on the optimal airway surface hydration state involving the presence of an airway surface layer (ASL). ASL is responsible for mechanical trapping of inhaled particles and pathogens by mucous component and for facilitating ciliary beating in presence of the periciliary layer. These facts have been confirmed in primary and secondary ciliary dyskinesia or cystic fibrosis. The former is related to the ciliary dysfunction as a consequence of defects in the ciliary structure based on the genetic mutations or inflammatory mediators and pH of the cilia environment, respectively (Rossman et al., 1984; Bisgaard and Pedersen, 1987; Clary-Meinesz et al., 1998; Gomperts et al., 2007; Grzela et al., 2013). The latter is caused by impaired epithelium hydration with high mucus viscosity as a result of mutations in the cystic fibrosis transmembrane conductance regulator (CFTR) gene. Over time, ciliary dysfunction leads to recurrent respiratory infections and consecutive decline in lung functions leading to decreased quality of patients’ life.

Secondary ciliary dyskinesia includes acquired defects of ciliary movement, which can be caused by viral or bacterial infection or by air pollutants. This may be evident in lung diseases such as bronchial asthma, and COPD. However, a direct relationship between ciliary dysfunction with ultrastructural abnormalities and disease severity was observed only in asthma subjects (Thomas et al., 2010). In COPD studies, controversial results were confirmed with the CBF discrepancy in response to cigarette smoke (Zhou et al., 2009; Cohen et al., 2009, Yaghi et al., 2012; Yaghi and Dolovich, 2016). Different CBF values related to the cilia length shortening due to autophagic mechanisms were also found by some researchers (Lam et al., 2013). Furthermore, the uncoordinated cilia function is not typical for moderate to severe stages of COPD, and the number of non-ciliated cells well correlates with the severity of the disease (Yaghi et al., 2012).

## Chronic Obstructive Pulmonary Disease

Chronic obstructive pulmonary disease (COPD) is a common, preventable, and treatable disease associated with chronic inflammation of the lung parenchyma and peripheral airways. This leads to persistent respiratory symptoms and progressive airflow limitation usually caused by significant exposure to tobacco smoke and airborne pollutants (GOLD Pocket Guide, 2019).

The pharmacotherapy of COPD is strictly individualized based on the disease severity, drug efficacy, drug tolerance, patient preferences, comorbidities, and potential drug interactions. Bronchodilators and anti-inflammatory medications are one of the cornerstones of COPD management. They reduce clinical symptoms, prevent the development of airflow limitation, lower the risk and severity of exacerbations, and improve the patients’ quality of life and exercise tolerance. Despite the relative effectivity of these agents, severe or very severe stages of the disease (FEV1 post-bronchodilator less than 50% predicted) can sometimes developed probably due to progression of inflammatory process.

Long-term inhaled treatment with corticosteroids (CS) in association with long-acting beta_2_ agonists (LABA) reduce but do not eliminate acute exacerbations of COPD. Methylxanthines have been accepted as being effective drugs for the treatment of COPD possessing both anti-inflammatory and bronchodilator activity in the same molecule. But findings about their non-selective mechanisms responsible for their relatively unfavourable safety profile have led to the development of novel therapeutic drugs with more selective properties.

### PDE/PDE Inhibitors and COPD

The discovery of phosphodiesterase isoenzymes (PDEs) along with increased understanding of their different function based on the tissue/cell specificity was a driving force for the development of PDE selective inhibitors for the treatment of various diseases including COPD. Up-to-date, 11 isoenzymes families of PDEs have been recognized (PDE1 - PDE11), of which PDE3 and PDE4 are the major cAMP-hydrolyzing enzymes identified in the smooth muscle. They are involved in the airway smooth muscle tone regulation and inflammatory cell activity. PDE1 and PDE5 are responsible for the cGMP-hydrolytic activity in airway and vascular smooth muscle (Torphy et al., 1993).

Roflumilast, the first selective PDE4 inhibitor approved for the therapy of COPD, is an anti-inflammatory agent that improves lung function in patients with moderate to severe COPD and reduces the risk of moderate to severe exacerbations when add on to bronchodilators (SABA, LABA), or inhaled CS. Although it is generally well tolerated, systemic effect of the drug is associated with the risk of diarrhea, nausea, weight loss, headaches, and suicide limiting its use and inhaled form if this drug with more favorable safety profile would be of benefit.

It is well known that most recommended drugs for COPD are given by inhalational route (GOLD Pocket Guide, 2019). It provides several significant advantages over systemic administration: lower effective dose than with systemic delivery, higher concentration of the drug reached in the airways, direct local delivery of the drug avoiding the systemic circulation and first pass effect in the liver. The benefits of local administration include fewer and less severe adverse effects, more selective actions, and direct interaction of the drug with ciliary epithelium. This makes the mucociliary clearance more efficient in removing of excess mucous from the lungs during pathological conditions, especially in case the drug has cilio-stimulatory properties.

Thus, mucus hypersecretion and chronic productive cough as the two characteristic features of COPD suggest the airway ciliary dysfunction and indicate the need for improvement of ciliary motility in the severe stages of this disease. At present, there are no specific therapies available to correct the ciliary dysfunction and the empiric treatment helps only to manage the consequences of dysfunctional cilia. Therefore, inhalational route of administration and focusing on mechanisms ameliorating the ciliary beating frequency (e.g., increased intracellular concentrations of cyclic nucleotides caused by PDE inhibition) might be of huge benefit (Milara et al., 2012; Kogiso et al., 2018; Zuo et al., 2018).

## PDE and Ciliary Motility

The activity of PDE proteins is dependent on cell type. In the human airway epithelial cells PDE4 activity is predominantly expressed in addition to lesser PDE1, PDE3, and PDE5 activity (**Table 1**) (Wright et al., 1998). The airway smooth muscle (ASM) human cells express both PDE3 and PDE4 enzymes (Beute et al., 2018). As described above, activity of PDE leads to relatively flexible changes in intracellular cAMP concentrations, which are involved in direct modulation of calcium mediated contractions of microtubular apparatus. As described in an older study by Tamaoki et al. (1989), the phosphodiesterase inhibition by 3-isobutyl-1-methylxanthine and the adenylate cyclase stimulation by forskolin led to increase in CBF in a dose-dependent manner and were accompanied by the increases in intracellular concentrations of cAMP. These results suggest that cAMP may accelerate mucociliary clearance through the activation of ciliary motility and that intracellular cAMP levels appear to be an important determinant for the lung mucociliary transport functions (Tamaoki et al., 1989). Similar results were observed by Schmid et al. (2006), who described dose-dependent, albeit similar and simultaneous increases in cAMP and ciliary beat frequency caused by forskolin administration on human airway epithelial cells. Therefore, an inhibition of PDEs, either non-selective or selective to mostly expressed isoforms in the airways, might be potentially useful in the modulation of CBF, either directly by changing the cAMP levels in cilia, or indirectly by influencing the allergic or other inflammation (Beute et al., 2018).

**Table 1:** PDE families expressed in ciliary epithelia with their selective inhibitors and references of papers describing modulatory effects on ciliary motility.

PDE family	Preference to cAMP or cGMP	Selective inhibitor	Ciliary motility modulation
PDE1A	Both	8MmIBMX	Kogiso et al., 2017
PDE3	Both	Milrinone	Cervin and Lindgren, 1998
PDE4	cAMP	Rolipram, Roflumilast	Cervin and Lindgren, 1998; Wohlsen et al., 2010; Milara et al., 2012
PDE5	cGMP	Zaprinast	Cervin and Lindgren, 1998

Chronic treatment with PDE inhibitors seems to have stronger effect on the ciliary beating since they do not directly increase the generation of cAMP, but decrease its breakdown. Therefore, their stronger action is expected in more progressed disease stages.

### Non-Selective PDE Inhibitors

There are several older studies focused on involvement of cyclic nucleotides in sperm ciliary impairment. Non-selective inhibitors of PDEs produce marked stimulation of cAMP dependent motility, but not all of them have equal efficacy. Caffeine was demonstrated to have a dose-dependent action with benefit at low to moderate doses in some human studies, whilst high-dose led to detrimental effects (Levin et al., 1981; Moussa, 1983; Jiang et al., 1984). Pentoxifylline (PTX), a potent vasodilating agent recommended for the treatment of leg pain caused by chronic occlusive arterial disease of the limbs, was studied as an artificial sperm movement enhancer (Słoczyńska et al., 2013; Aifantis et al., 2019). Its effect on sperm motility was confirmed in several *in vivo* studies with significantly increased sperm motility in men with oligoasthenoteratozoospermia (OAT) or *in vitro* studies during a vitrification program without adverse effects on sperm DNA or chromatin integrity (Stanic et al., 2002; Moslemi Mehni et al., 2014; Nabi et al., 2017). However, the results of PTX treatment in terms of assisted reproductive technologies outcomes are not consistent. This is due to the treatment failure of PTX in improvement of ejaculate parameters including the motility (Maier et al., 1994). In *in vitro* conditions, PTX significantly enhanced the viability of sperm of infertile OAT males with no significant effect on its motility (Ghasemzadeh et al., 2016).

Theophylline has been confirmed to have a protective impact on sperm motility during freezing procedures (Ebner et al., 2011; Gorji et al., 2018). However, teratogenic potential of methylxanthines is a limiting factor of their clinical use in improving sperm motility and fertilizing ability (Fujii and Nishimura, 1969; Tucci and Skalko, 1978; Friedman et al., 1979; Basnet et al., 2017). Moreover narrow therapeutic index and non-selective mechanism of action with high risk of systemic adverse effects favors other drugs over methylxanthines.

Similarly to sperm cilia, tracheal ciliary epithelium is controlled by intracellular cAMP levels. In a recent animal study was confirmed that the ciliary beat frequency in tracheal epithelial cells is significantly higher, when ovalbumin-sensitized guinea pigs were treated with systemic theophylline at the dose of 10 mg/kg/day (Kazimierová et al., 2015). This suggests direct involvement of PDE inhibition in ameliorating the impaired defense mechanisms caused by sensitization and challenging the animals with ovalbumin, used to mimic the asthma-like conditions (airway hyperresponsiveness associated with eosinophilic inflammation). However, this study did not reveal the exact involvement of PDE inhibition in direct influence on cilia or indirect effects caused by anti-inflammatory and immunomodulation action of theophylline. Therefore, selective PDE inhibitors responsible for inhibiting single PDE isoforms expressed in ciliary epithelial cells need to be tested.

### Selective PDE1 Inhibitors

**Figure 2:**
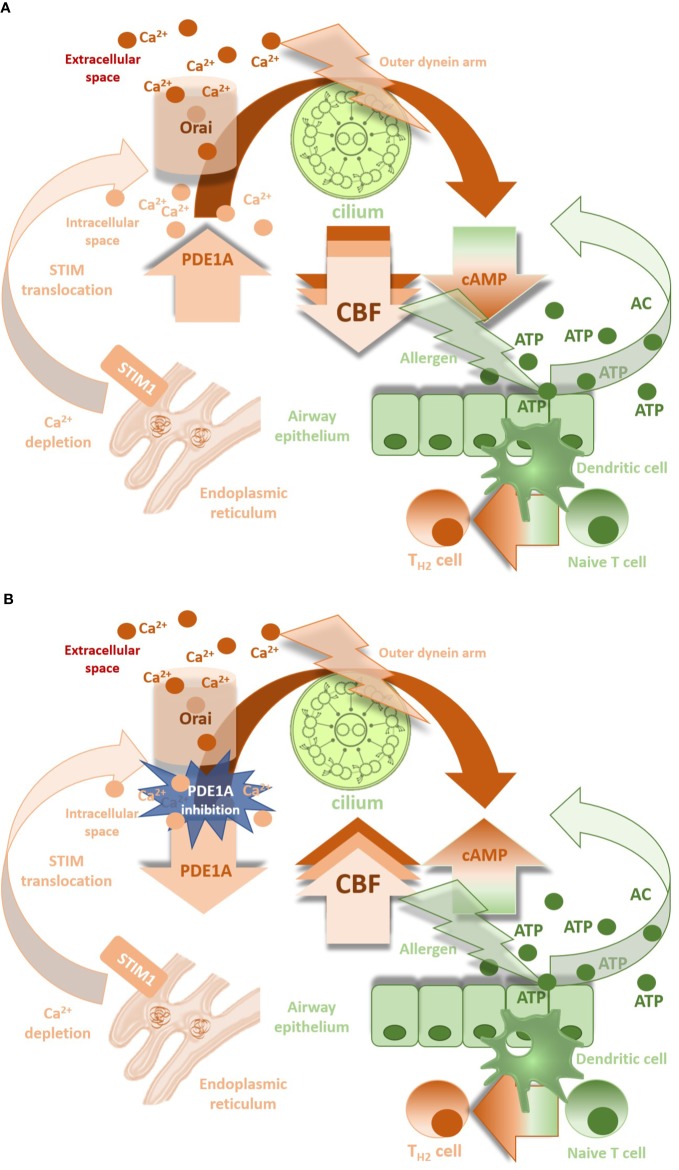
**(A)**: CBF down-regulation by PDE1A in presence of Ca^2+^ ions. **(B)** Involvement of PDE1A inhibition in CBF increase (CBF, ciliary beat frequency; PDE1A, phosphodiesterase 1A; cAMP, cyclic 3′, 5′-adenosine monophosphate; Orai, calcium release-activated calcium channel involved in T-lymphocytes activation; STIM1, stromal interaction molecule 1 - calcium sensor in endoplasmic reticulum).

The experimental study of Kogiso et al. (2017) has recently confirmed the expression of PDE1A by immune-electron microscopy methods in the cilia and the cell body of lung airway ciliary cells. Ca^2+^/calmodulin-dependent PDE1A was detected on the nine doublet tubules ring and outside the ring in the lung airway cilium, where the outer dynein arm functions. PDE1A modulates CBF in lung cells in a Ca^2+^ dependent manner (**Figures 2A, B**). PDE1A inhibition induced by low intracellular Ca^2+^ enhances CBF due to cAMP accumulation (Kogiso et al., 2018).

PDE1C isoform was found in the mitochondria of cell body, but not in the cilia. Similar results were observed in motile sperms of mice (Vasta et al., 2005) as well as in human spermatozoa (Lefievre et al., 2002), where a new isoform of PDE1A was detected.

Despite of these observations, there is currently only limited data about efficacy of PDE1 inhibitors in respiratory diseases. Vinpocetin, a selective PDE1 inhibitor used for increasing learning and memory due to its vasodilating effect in cerebral circulation caused by cGMP increase, was shown to have mild antitussive and bronchodilating action in healthy and ovalbumin sensitized guinea pigs (Mokry and Nosalova, 2011). However, no information about its effects on CBF is available.

### Selective PDE4 Inhibitors

As mentioned above, the airway smooth muscle (ASM) human cells as well as epithelial cells express PDE4 enzymes (Beute et al., 2018; Mokry et al., 2018). This is consistent with the findings of *in vitro* experiments showing that ASM human cells are primarily sensitive to cilostamid (PDE3 inhibitor), while both ASM cells and human non-polarized bronchial epithelial (16HBE 14o) cells are responding to PDE4 inhibition by roflumilast. Moreover, selective PDE4 inhibition was found to induce an effective reversion in the CBF down-regulation induced by cigarette exposure in *ex vivo* conditions under which expression of both PDE3 and PDE4 enzymes is dominant (Milara et al., 2012; Zuo et al., 2018). Furthermore, LABA enables roflumilast (selective PDE4 inhibitor) ability to stimulate CBF in cigarette smoke exposed cells, while roflumilast itself does it only in limited manner, as demonstrated by Schmid et al. (2015). They showed that roflumilast can rescue smoke-induced mucociliary dysfunction by reversing decreased cystic fibrosis transmembrane conductance regulator (CFTR) activity, augmenting airway surface liquid volume, and stimulating CBF, the latter particularly in combination with formoterol. Of the 11 PDE families, PDE3 and PDE4 have been shown to regulate cAMP-stimulated CFTR, thus PDE4 inhibitors can potentiate the effect of CFTR correctors due to regulation of ion and water transport across epithelia (Blanchard et al., 2014). Workman and Cohen (2014) examined 229 substances based on their effects on CBF. Among PDE4 inhibitors, roflumilast, roflumilast-N-oxide, and ibudilast increased CBF, whereas theophylline as a non-selective PDE inhibitor decreased it, probably due to antagonistic action on adenosine A_2B_ receptors (Allen-Gipson et al., 2011). Furthermore, most of tested glucocorticosteroids and anticholinergics led to decrease of CBF, suggesting a benefit of co-administration of PDE4 inhibitors with these groups of drugs to prevent worsening of expectoration in COPD patients (Workman and Cohen, 2014).

### Dual PDE3/4 Inhibitors

Dual PDE3/4 inhibitors represent another strategy of potential pharmaco-therapeutic agents for the treatment of respiratory diseases because of their mixed bronchodilator/anti-inflammatory effects and enhanced efficacy in a single molecule compared to inhibition of only PDE3 or PDE4. Their benefit involves efficacy both in acute and long-lasting bronchodilation along with anti-inflammatory properties (Singh et al., 2018). Clinical use of a inhaled bifunctional PDE3/4 inhibitor (ensifentrine/RPL554) in respiratory diseases is currently limited to a few studies, including bronchial asthma, in which mixed PDE3/4 inhibition has a beneficial effect comparable with salbutamol but avoiding characteristic systemic adverse effects of beta_2_ agonists (Bjermer et al., 2019). In a recent study, RPL554 increased CBF in primary human bronchial epithelial cells, suggesting that RPL554 may increase mucociliary clearance through stimulation of CFTR and increasing CBF and, thus, could provide a novel therapeutic option for, e.g., cystic fibrosis (Turner et al., 2016). Ensifentrine has recently entered clinical phase II of drug testing, in order to demonstrate its benefits as an add-on therapy in patients for the treatment of acute exacerbations of COPD or for the regular maintenance treatment of patients either alone, or on top of existing drug classes (Cazzola et al., 2018). However, at present the most intriguing perspective is linked to its possible use in the treatment of cystic fibrosis, also considering the lack of valid therapeutic options for this disease (Cazzola et al., 2019).

## Other Drugs with Cilio-Stimulatory Properties (Mucokinetics) Used for COPD and Asthma Therapy

### Beta_2_ Agonists

The mucokinetic agents may influence mucociliary clearance directly by acting on the cilia. There is evidence that bronchodilators SABA and LABA improve beating kinematics *via* increase in the CBF in *in vitro* conditions (Devalia et al., 1992; Kanthakumar et al., 1994; Frohock et al., 2002; Pappova et al., 2017). However, in patients with moderate and severe bronchial asthma, the CBF significantly declines despite treatment with long-acting bronchodilators (Thomas et al., 2010). The question is to what extent the CBF is influenced by direct effect of the drug in conditions of chronic inflammation and epithelium remodeling. Kogiso et al. (2018) demonstrated that increase in the intracellular Ca^2+^ concentration stimulates PDE1A leading to degradation of cAMP and to suppression of the procaterol-stimulated CBF increase. Ca^2+^ influx represents a motor for PDE1A most likely *via* calcium release-activated ion channels (CRAC), whilst Ca^2+^ released from intracellular stores plays only little or no role (Zagoory et al., 2002; Goraya et al., 2004; Joskova et al., 2016). CRAC ion channels belong to the group of store operated ion channels and become active when Ca^2+^ intracellular sources are depleted. It seems that CRAC channel opening is involved in a dual mechanism: extracellular Ca^2+^ enables the refilling of endoplasmic reticulum Ca^2+^ stores (Prakriya, 2009) with Ca^2+^-mediated cilio-stimulatory impact, but also links with cAMP-dependent PDE1A activities. The overall change in ciliary beating is dependent on the balance of Ca^2+-^signal and cAMP-signal (Kogiso et al., 2018) with dominant response of the latter in pathological conditions due to the cAMP breakdown (**Figure 2A**). This hypothesis has been confirmed in our experiments with significant decrease in the CBF during physiological conditions, when CRAC channel blocker was used, whilst different ciliary response seen in airway allergic inflammation in ovalbumin-sensitized guinea pigs (Joskova et al., 2016). Upregulation of STIM1 and Orai1 proteins, the molecular components of CRAC ion channels in inflammatory conditions can explain our findings (Spinelli et al., 2012). Moreover, high levels of ATP associated with tissue damage or inflammation provides a substrate for soluble adenylyl cyclase (sAC) and generation of more cAMP for being PDE active to slow down the ciliary beating. This explains potential benefit of selective PDE1A inhibition in increasing the CBF (**Figure 2B**). Furthermore, the closing connexin/pannexin hemichannels that release ATP and decreasing intracellular Ca^2+^ levels may also suppress the CBF (Schmid et al., 2014; Shishikura et al., 2016; Droguett et al., 2017; Zhou et al., 2019).

Similar results were obtained in *in vitro* studies modelling COPD; the increase in the CBF induced by beta_2_ agonist was abolished by up-regulation of IL-8 in response to cigarette smoke. Thus, it is likely that smokers may be more resistant to the cilio-stimulatory effects of beta_2_ agonists (Allen-Gipson et al., 2004).

### Anticholinergic Drugs

Although it is generally believed that acetylcholine increases CBF *via* muscarinic receptors (Wanner et al., 1996; Salathe et al., 1997; Zagoory et al., 2001), anticholinergic agents exert cilio-inhibitory effects of different intensities in experimental studies (Pavia et al., 1979; Wanner, 1986). Muscarinic receptor antagonists provide benefit in COPD by blocking cholinergic tone and bronchodilation; however, there is no clinical evidence about their effect on the ciliary beating in such conditions. M_3_ receptors are involved in the control of ciliary beat frequency, whereas M_2_ receptors play an opposite role and prevent cilia-excitation initiated by M_1_ receptors as well as by non-muscarinic stimuli such as ATP. However, M_2_ receptors are not detectable in the epithelial cells and are found only in neighboring cells (Klein et al., 2009).

### Glucocorticosteroids

In animal studies, chronic inhalation of budesonide significantly lowered the CBF using a 21-days model of ovalbumin-induced allergic airway inflammation, whilst non-significant changes of this parameter have been documented in the 28-days model (Pappová et al., 2016) despite decreased level of IL-13 determined in bronchoalveolar lavage fluid and significant anti-inflammatory effect. In patients with COPD, acute inhalation of beclomethasone dipropionate did not affect mucociliary clearance rates (Fazio and Lafortuna, 1986; Guleria et al., 2003). There are no studies and data on the long-term effects of chronic therapy with corticosteroids on the ciliary beat frequency and mucociliary clearance. However, a long-term inhalation of steroids in severe asthmatic patients leads to reduced epithelium damage and decreased non-ciliated areas in the ciliated epithelium (Lundgren et al., 1988). This may be consistent with the experimental finding that cilio-stimulatory effects of LABA increase in presence of inhaled corticosteroids and the fact that ICS-LABA therapy significantly reduced the rate of exacerbations (Pappová et al., 2016; GOLD Pocket Guide, 2019; GINA Pocket Guide, 2019).

### Antileukotriens

Leukotrienes C_4_ and D_4_ as lipid mediators (products of arachidonic acid degradation by 5-lipooxygenase) increase the ciliary beat frequency in human upper airway mucosa *in vitro* (Cyrus et al., 1998). Moreover, experimental findings suggest that LTD_4_ exerts cilio-excitatory effects in human, guinea pig, and rat respiratory mucosa with impaired cilia orientation and mucus production (Joki et al., 1996).

Leukotriene modifiers could contribute to cilio-modulatory effect due to both time- and dose- dependent cilio-stimulatory effect of montelukast in human sinonasal epithelial cultures (Uz et al., 2014); however, these experiments have not been yet performed in inflammatory conditions.

### Drug Excipients and the Ciliary Beating Frequency

The cilio-inhibitory or cilio-static effect of preservatives and absorption enhancers used as drug excipients should also be taken in account. Despite their effectiveness in drug absorption and prevention of microbial contamination, they may interfere with mucociliary clearance and ciliary function (Merkus et al., 2001; Jiao and Zhang, 2019).

## Conclusions

Airway epithelial PDE isoforms could be considered as important targets for the development of new inhaled mucokinetic agents. Their inhibition represents a viable therapeutic approach to enhance mucociliary clearance in respiratory diseases. The major mechanisms involved in an overall improvement of mucociliary clearance in respiratory disease due to PDE1, PDE4 or PDE3/4 inhibitors include:

- increase in the CBF to allow a removal of mucus more effectively from the lungs (PDE1, PDE4, PDE3/4 inhibitors);
- better epithelium hydration lowering the mucus viscosity (PDE4, PDE3/4 inhibitors);
- anti-inflammatory effect (PDE4 and PDE3/4 inhibitors);
- bronchodilator effects (PDE3, PDE3/4 inhibitors);
- relatively few systemic adverse effects when delivered *via* the inhaled route.

Furthermore, PDE1A inhibitors potentiate the cilia-stimulatory effects of beta_2_ agonist mediated by cAMP accumulation, whilst PDE4 inhibitors become effective in the presence of LABA to stimulate ciliary beating. This makes the mucociliary clearance in pathological airway conditions more effective.

Dual PDE3/4 inhibitors could provide additional benefits and show more rapid response when taken with beta_2_ agonist; however, their cilia-stimulatory effect is probably mediated predominantly by PDE4 inhibition.

In summary, PDE inhibitors besides their anti-inflammatory and bronchodilator properties can prevent recurrent infection, persistent inflammation, and decline in lung functions *via* direct cilia-stimulatory response, when cilia dysfunction in lung diseases has developed. Further research is required to provide evidence of the effectiveness and feasibility of the inhaled selective or bifunctional PDE inhibitors with respect to their cilia-modulating properties.

## Author Contributions

MJ wrote the first draft of the manuscript. JM wrote sections of the manuscript. MJ, JM, and SF contributed to the manuscript revision, read, and approved the submitted version.

## Funding

Supported by grants VEGA 1/0160/17, VEGA 1/0356/18, VEGA 1/0253/19, and APVV-18-0084, and by the project “Measurement of Respiratory Epithelium Cilium Kinematics” (ITMS 2622022019).

## Conflict of Interest

The authors declare that the research was conducted in the absence of any commercial or financial relationships that could be construed as a potential conflict of interest.

## References

[B1] Aifantis K. E. Shrivastava S. Pelidou S.-H. Ngan A. H. W. Baloyannis S. I. (2019). Relating the blood-thinning effect of pentoxifylline to the reduction in the elastic modulus of human red blood cells: an in vivo study. Biomater. Sci. 7, 2545–2551. 10.1039/c8bm01691g 30973560

[B2] Allen-Gipson D. S. Romberger D. J. Forget M. A. May K. L. Sisson J. H. Wyatt T. A. (2004). IL-8 inhibits isoproterenol-stimulated ciliary beat frequency in bovine bronchial epithelial cells. J. Aerosol. Med. 17, 107–115. 10.1089/0894268041457138 15294060

[B3] Allen-Gipson D. S. Blackburn M. R. Schneider D. J. Thang H. Bluitt D. L. Jarrell J. C. (2011). Adenosine activation of A2B receptor(s) is essential for stimulated epithelial ciliary motility and clearance. Am. J. Physiol. Lung Cell. Mol. Physiol. 301, L171–L180. 10.1152/ajplung.00203.2010 21622845 PMC3154627

[B4] Andrade Y. N. Fernandes J. Vazquez E. Fernandez-Fernandez J. M. Arniges M. Sanchez T. M. (2005). TRPV4 channel is involved in the coupling of fluid viscosity changes to epithelial ciliary activity. J. Cell Biol. 168, 869–874. 10.1083/jcb.200409070 15753126 PMC2171792

[B5] Bailey K. L. LeVan T. D. Yanov D. A. Pavlik J. A. DeVasure J. M. Sisson J. H. (2012). Non-typeable Haemophilus influenzae decreases cilia beating via protein kinase Cϵ. Respir. Res. 13, 49. 10.1186/1465-9921-13-49 22712879 PMC3487807

[B6] Basnet R. M. Zizioli D. Guarienti M. Finazzi D. Memo M. (2017). Methylxanthines induce structural and functional alterations of the cardiac system in zebrafish embryos. BMC Pharmacol. Toxicol. 18, 72. 10.1186/s40360-017-0179-9 29141695 PMC5688754

[B7] Beute J. Lukkes M. Koekoek E. P. Nastiti H. Ganesh K. de Bruijn M. J. (2018). A pathophysiological role of PDE3 in allergic airway inflammation. JCI Insight. 3, 94888. 10.1172/jci.insight.94888 29367458 PMC5821178

[B8] Bisgaard H. Pedersen M. (1987). SRS-A leukotrienes decrease the activity of human respiratory cilia. Clin. Allergy 17, 95–103. 10.1111/j.1365-2222.1987.tb02326.x 3581463

[B9] Bjermer L. Abbott-Banner K. Newman K. (2019). Efficacy and safety of a first-in-class inhaled PDE3/4 inhibitor (ensifentrine) vs salbutamol in asthma. Pulm. Pharmacol. Ther. 58, 101814. 10.1016/j.pupt.2019.101814 31202957

[B10] Blanchard E. Zlock L. Lao A. Mika D. Namkung W. Xie M. (2014). Anchored PDE4 regulates chloride conductance in wild-type and ΔF508-CFTR human airway epithelia. FASEB J. 28, 791–801. 10.1096/fj.13-240861 24200884 PMC3898646

[B11] Braga P. C. Dal Sasso M. Sala M. T. Gianelle V. (1999). Effects of erdosteine and its metabolites on bacterial adhesiveness. Arzneimittelforschung 49, 344–350. 10.1055/s-0031-1300425 10337454

[B12] Cano D. A. Sekine S. Hebrok M. (2006). Primary cilia deletion in pancreatic epithelial cells results in cyst formation and pancreatitis. Gastroenterology 131, 1856–1869. 10.1053/j.gastro.2006.10.050 17123526

[B13] Cazzola M. Page C. Calzetta L. Matera M. G. (2018). Ensifentrine (RPL554): an inhaled ‘bifunctional’ dual PDE3/4 inhibitor for the treatment of asthma and chronic obstructive pulmonary disease. Pharm. Pat. Anal. 7, 249–257. 10.4155/ppa-2018-0030 30657422

[B14] Cazzola M. Calzetta L. Rogliani P. Matera M. G. (2019). Ensifentrine (RPL554): an investigational PDE3/4 inhibitor for the treatment of COPD. Expert Opin. Investig. Drugs 28, 827–833. 10.1080/13543784.2019.1661990 31474120

[B15] Cervin A. Lindgren S. (1998). The effect of selective phosphodiesterase inhibitors on mucociliary activity in the upper and lower airways in vitro. Auris Nasus Larynx. 25, 269–276. 10.1016/s0385-8146(98)00010-8 9799993

[B16] Clary-Meinesz C. Mouroux J. Cosson J. Huitorel P. Blaive B. (1998). Influence of external pH on ciliary beat frequency in human bronchi and bronchioles. Eur. Respir. J. 11, 330–333. 10.1183/09031936.98.11020330 9551733

[B17] Cohen N. A. Zhang S. Sharp D. B. Tamashiro E. Chen B. Sorscher E. J. (2009). Cigarette smoke condensate inhibits transepithelial chloride transport and ciliary beat frequency. Laryngoscope 119, 2269e74. 10.1002/lary.20223 19418539

[B18] Cyrus C. B. Yang B. McCaffrey T. V. (1998). Leukotrienes C4 and D4 increase the ciliary beat frequency in human upper airway mucosa in vitro. Otolaryngol. Head Neck Surg. 118, 472–477. 10.1177/019459989811800407 9560097

[B19] Devalia J. L. Sapsford R. J. Rusznak C. Toumbis M. J. Davies R. J. (1992). The effects of salmeterol and salbutamol on ciliary beat frequency of cultured human bronchial epithelial cells, in vitro. Pulm. Pharmacol. 5, 257–263. 10.1016/0952-0600(92)90068-r 1362105

[B20] Droguett K. Rios M. Carreño D. V. Navarrete C. Fuentes C. Villalón M. (2017). An autocrine ATP release mechanism regulates basal ciliary activity in airway epithelium. J. Physiol. 595, 4755–4767. 10.1113/JP273996 28422293 PMC5509870

[B21] Ebner Th. Tews G. Mayer R. B. Ziehr S. Arzt W. Costamoling W. (2011). Pharmacological stimulation of sperm motility in frozen and thawed testicular sperm using the dimethylxanthine theophylline. Fertil. Steril. 96, 1331–1336. 10.1016/j.fertnstert.2011.08.041 21962960

[B22] Falk N. Lösl M. Schröder N. Gießl A. (2015). Specialized Cilia in Mammalian Sensory Systems. Cells 4, 500–519. 10.3390/cells4030500 26378583 PMC4588048

[B23] Fazio F. Lafortuna C. L. (1986). Beclomethasone Dipropionate Does Not Affect Mucociliary Clearance in Patients with Chronic Obstructive Lung Disease. Respiration 50, 62–65. 10.1159/000194908 3726286

[B24] Feldman C. Anderson R. Cockeran R. Mitchell T. Cole P. Wilson R. (2002). The effects of pneumolysin and hydrogen peroxide, alone and in combination, on human ciliated epithelium in vitro. Respir. Med. 96, 580–585. 10.1053/rmed.2002.1316 12195838

[B25] Fraňová S. Kazimierová I. Pappová L. Molitorisová M. Jošková M. Šutovská M. (2019). The effect of erdosteine on airway defence mechanisms and inflammatory cytokines in the settings of allergic inflammation. Pulm. Pharmacol. Ther. 54, 60–67. 10.1016/j.pupt.2018.11.006 30502381

[B26] Friedman L. Weinberger M. A. Farber T. M. Moreland F. M. Peters E. L. Gilmore C. E. (1979). Testicular atrophy and impaired spermatogenesis in rats fed high levels of the methylxanthines caffeine, theobromine, or theophylline. J. Environ. Pathol. Toxicol. 2, 687–706. 422930

[B27] Frohock J. I. Wijkstrom-Frei C. Salathe M. (2002). Effects of albuterol enantiomers on ciliary beat frequency in in ovine tracheal epithelial cells. J. Appl. Physiol. 92, 2396–2402. 10.1152/japplphysiol.00755.2001 12015353

[B28] Fujii T. Nishimura H. (1969). Teratogenic actions of some methylated xanthines in mice. Okajimas Folia Anat. Jpn. 46, 167–175. 10.2535/ofaj1936.46.4_167 5394617

[B29] Ghasemzadeh A. Karkon-Shayan F. Yousefzadeh S. Naghavi-Behzad M. Hamdi K. (2016). Study of pentoxifylline effects on motility and viability of spermatozoa from infertile asthenozoospermic males. Niger. Med. J. 57, 324–328. 10.4103/0300-1652.193857 27942099 PMC5126744

[B30] GINA Pocket Guide (2019). “Pocket guide for asthma management and prevention for adults and Children older than 5 years,” in A pocket guide for Health Professionals. Updated https://ginasthma.org/wp-content/uploads/2019/04/GINA-2019-main-Pocket-Guide-wms.pdf.

[B31] GOLD Pocket Guide (2019). “Global Initiative for Chronic Obstructive Lung Disease. Pocket guide to COPD Diagnosis, Management, and Prevention,” in A Guide for Health Care Professionals. Global Initiative for Chronic Obstructive Lung Disease, Inc. Report. https://goldcopd.org/wp-content/uploads/2018/11/GOLD-2019-POCKET-GUIDE-FINAL_WMS.pdf.

[B32] Gomperts B. N. Kim L. J. Flaherty S. A. Hackett B. P. (2007). IL-13 Regulates Cilia Loss and foxj1 Expression in Human Airway Epithelium. Am. J. Respir. Cell Mol. Biol. 37, 339–346. 10.1165/rcmb.2006-0400OC 17541011 PMC2720122

[B33] Goraya T. A. Masada N. Ciruela A. Cooper D. M. (2004). Sustained entry of Ca2+ is required to activate Ca2+-calmodulin-dependent phosphodiesterase 1A. J. Biol. Chem. 279(39), 40494–40504. 15272012 10.1074/jbc.M313441200

[B34] Gorji E. Farsi M. M. Khafri S. Shafi H. (2018). Analysis of the impact of cryopreservation and theophylline on motility of sperm. Middle East Fertil. Soc J. 23, 98–102. 10.1016/j.mefs.2017.09.002

[B35] Grati M. Chakchouk I. Ma Q. Bensaid M. Desmidt A. Turki N. (2015). A missense mutation in DCDC2 causes human recessive deafness DFNB66, likely by interfering with sensory hair cell and supporting cell cilia length regulation. Hum. Mol. Genet. 24, 2482–2491. 10.1093/hmg/ddv009 25601850 PMC4383862

[B36] Grzela K. Zagórska W. Jankowska-Steifer E. Grzela T. (2013). Chronic inflammation in the respiratory tract and ciliary dyskinesia. Centr. Eur. J. Immunol. 38, 122–128. 10.5114/ceji.2013.34369

[B37] Guleria R. Singh T. R. Sinha S. Padhy K. Gupta K. Pande J. N. (2003). Effect of single inhalation of a salbutamol, ipratropium bromide and beclomethasone on mucociliary clearance in patients with chronic obstructive airway disease. Indian J. Chest Dis. Allied Sci. 45, 241–246. 12962458

[B38] Hou X. Mrug M. Yoder B. K. Lefkowitz E. J. Kremmidiotis G. D’Eustachio P. (2002). Cystin, a novel cilia-associated protein, is disrupted in the cpk mouse model of polycystic kidney disease. J. Clin. Invest. 109, 533–540. 10.1172/JCI14099 11854326 PMC150876

[B39] Jiang C. S. Kilfeather S. A. Pearson R. M. Turner P. (1984). The stimulatory effects of caffeine, theophyline, lysine-theophylline and 3-isobutyl-1-methylxanthine on human sperm motility. Br. J. Clin. Pharmacol. 18, 258. 10.1111/j.1365-2125.1984.tb02466.x 6207849 PMC1463533

[B40] Jiao J. Zhang L. (2019). Influence of Intranasal Drugs on Human Nasal Mucociliary Clearance and Ciliary Beat Frequency. Allergy Asthma Immunol. Res. 11, 306–319. 10.4168/aair.2019.11.3.306 30912321 PMC6439188

[B41] Joki S. Saano V. Koskela T. Toskala E. Bray M. A. Nuutinen J. (1996). Effect of leukotriene D4 on ciliary activity in human, guinea-pig and rat respiratory mucosa. Pulm. Pharmacol. 9, 231–238. 10.1006/pulp.1996.0029 9160411

[B42] Jorissen M. Willems T. Van der Schueren B. (2000). Ciliary function analysis for the diagnosis of primary ciliary dyskinesia: advantages of ciliogenesis in culture. Acta Otolaryngol. 120, 291–295. 10.1080/000164800750001116 11603792

[B43] Joskova M. Sutovska M. Durdik P. Koniar D. Hargas L. Banovcin P. (2016). The Role of Ion Channels to Regulate Airway Ciliary Beat Frequency During Allergic Inflammation. Adv. Exp. Med. Biol. 921, 27–35. 10.1007/5584_2016_247 27369295

[B44] Kanthakumar K. Cundell D. R. Johnson M. Wills P. J. Taylor G. W. Cole P. J. (1994). Effect of salmeterol on human nasal epithelial cell ciliary beating: inhibition of the ciliotoxin, pyocyanin. Br. J. Pharmacol. 112, 493–498. 10.1111/j.1476-5381.1994.tb13100.x 7915610 PMC1910368

[B45] Kazimierová I. Jošková M. Pecháňová O. Šutovská M. Fraňová S. (2015). Effects of Provinol and Its Combinations with Clinically Used Antiasthmatics on Airway Defense Mechanisms in Experimental Allergic Asthma. Adv. Exp. Med. Biol. 7, 27–34. 10.1007/5584_2014_75 25315622

[B46] King S. M. (2016). Axonemal Dynein Arms. Cold Spring Harb. Perspect. Biol. 8, a028100. 10.1101/cshperspect.a028100 PMC5088525 27527589

[B47] Klein M. K. Haberberger R. V. Hartmann P. Faulhammer P. Lips K. S. Krain B. (2009). Muscarinic receptor subtypes in cilia-driven transport and airway epithelial development. Eur. Respir. J. 33, 1113–1121. 10.1183/09031936.00015108 19213795 PMC3895332

[B48] Kogiso H. Hosogi S. Ikeuchi Y. Tanaka S. Shimamoto C. H. Matsumura H. (2017). A low [Ca^2+^]_i_-induced enhancement of cAMP-activated ciliary beating by PDE1A inhibition in mouse airway cilia. Pflugers Arch. – Eur. J. Physiol. 469, 1215–1227. 10.1007/s00424-017-1988-9 28477148

[B49] Kogiso H. Hosogi S. Ikeuchi Y. Tanaka S. Inui T. Marunaka Y. (2018). [Ca2+]i modulation of cAMP-stimulated ciliary beat frequency via PDE1 in airway ciliary cells of mice. Exp. Physiol. 103, 381–390. 10.1113/EP086681 29282782

[B50] Kulaga H. M. Leitch C. C. Eichers E. R. Badano J. L. Lesemann A. Hoskins B. E. (2004). Loss of BBS proteins causes anosmia in humans and defects in olfactory cilia structure and function in the mouse. Nat. Genet. 36, 994–998. 10.1038/ng1418 15322545

[B51] Kultgen P. L. Byrd S. K. Ostrowski L. E. Milgram S. L. (2002). Characterization of an A-kinase anchoring protein in human ciliary axonemes. Mol. Biol. Cell. 13, 4156–4166. 10.1091/mbc.e02-07-0391 12475942 PMC138623

[B52] Lam H. C. Cloonan S. M. Bhashyam A. R. Haspel J. A. Singh A. Sathirapongsasuti J. F. (2013). Histone deacetylase 6-mediated selective autophagy regulates COPD-associated cilia dysfunction. J. Clin. Invest. 123, 5212–5230. 10.1172/JCI69636 24200693 PMC3859407

[B53] Lansley A. B. Sanderson M. J. Dirksen E. R. (1992). Control of the beat cycle of respiratory tract cilia by Ca2+ and cAMP. Am. J. Physiol. 263, L232–L242. 10.1152/ajplung.1992.263.2.L232 1325130

[B54] Lefievre L. de Lamirande E. Gagnon C. (2002). Presence of cyclic nucleotide phosphodiesterases PDE1A, existing as a stable complex with calmodulin, and PDE3A in human spermatozoa. Biol. Reprod. 67, 423–430. 10.1095/biolreprod67.2.423 12135876

[B55] Levin R. M. Greenberg S. H. Wein A. J. (1981). Quantitative analysis of the effect of caffeine on sperm motility and cyclic adenosine 3′,5′-monophosphate (AMP) phosphodiesterase. Fertil. Steril. 36, 798. 10.1016/s0015-0282(16)45928-5 6273241

[B56] Lorenzo I. M. Liedtke W. Sanderson M. J. Valverde M. A. (2008). TRPV4 channel participates in receptor-operated calcium entry and ciliary beat frequency regulation in mouse airway epithelial cells. Proc. Natl. Acad. Sci. U. S. A. 105, 12611–12616. 10.1073/pnas.0803970105 18719094 PMC2527959

[B57] Lundgren R. Soderberg M. Horstedt P. Stenling R. (1988). Morphological studies of bronchial mucosal biopsies from asthmatics before and after ten years of treatment with inhaled steroids. Eur. Respir. J. 1, 883–889. 3224689

[B58] Ma W. Silberberg S. D. Priel Z. (2002). Distinct axonemal processes underlie spontaneous and stimulated airway ciliary activity. J. Gen. Physiol. 120, 875–885. 10.1085/jgp.20028695 12451055 PMC2229561

[B59] Maier U. Szabo N. Ludvik G. (1994). Oral Pentoxifylline in Therapy-Resistant Idiopathic OAT Syndrome. Arch. Androl. 33, 59–62. 10.3109/01485019408987803 7979810

[B60] Merkus P. Romeijn S. G. Verhoef J. C. Merkus F. W. H. M. Schouwenburg P. F. (2001). Classification of Cilio-Inhibiting Effects of Nasal Drugs. Laryngoscope. 111, 595–602. 10.1097/00005537-200104000-00008 11359126

[B61] Milara J. Armengot M. Bañuls P. Tenor H. Beume R. Artigues E. (2012). Roflumilast N-oxide, a PDE4 inhibitor, improves cilia motility and ciliated human bronchial epithelial cells compromised by cigarette smoke in vitro. Br. J. Pharmacol. 166, 2243–2262. 10.1111/j.1476-5381.2012.01929.x 22385203 PMC3448891

[B62] Miyake K. Kaise T. Hosoe H. Akuta K. Manabe H. Ohmori K. (1999). The effect of erdosteine and its active metabolite on reactive oxygen species production by inflammatory cells. Inflamm. Res. 48, 205–209. 10.1007/s000110050447 10344471

[B63] Mokry J. Nosalova G. (2011). The influence of the PDE inhibitors on cough reflex in guinea pigs. Bratisl. Lek. Listy. 112, 131–135. 21452764

[B64] Mokry J. Mokra D. Nosalova G. Beharkova M. Feherova Z. (2008). Influence of selective inhibitors of phosphodiesterase 3 and 4 on cough and airway reactivity. J. Physiol. Pharmacol. 59 (Suppl 6), 473–482. 19218671

[B65] Mokry J. Urbanova A. Kertys M. Mokra D. (2018). Inhibitors of phosphodiesterases in the treatment of cough. Respir. Physiol. Neurobiol. 257, 107–114. 10.1016/j.resp.2018.01.008 29337269

[B66] Moslemi Mehni N. Ketabchi A. A. Hosseini E. (2014). Combination effect of Pentoxifylline and L-carnitine on idiopathic oligoasthenoteratozoospermia. Iran J. Reprod. Med. 12, 817–824. 25709639 PMC4330662

[B67] Moussa M. M. (1983). Caffeine and sperm motility. Fertil. Steril. 39, 845–848. 10.1016/S0015-0282(16)47128-1 6343128

[B68] Murga-Zamalloa C. A. Atkins S. J. Peranen J. Swaroop A. Khanna H. (2010). Interaction of retinitis pigmentosa gtpase regulator (rpgr) with RAB8A gtpase: Implications for cilia dysfunction and photoreceptor degeneration. Hum. Mol. Genet. 19, 3591–3598. 10.1093/hmg/ddq275 20631154 PMC2928130

[B69] Nabi A. Khalili M. A. Fesahat F. Talebi A. Ghasemi-Esmailabad S. (2017). Pentoxifylline increase sperm motility in devitrified spermatozoa from asthenozoospermic patient without damage chromatin and DNA integrity. Cryobiology 76, 59–64. 10.1016/j.cryobiol.2017.04.008 28455156

[B70] Pappová L. Jošková M. Kazimierová I. Šutovská M. Fraňová S. (2016). Combination Therapy with Budesonide and Salmeterol in Experimental Allergic Inflammation. Adv. Exp. Med. Biol. 935, 25–34. 10.1007/5584_2016_24 27329088

[B71] Pappová L. Kazimierová I. Jošková M. Šutovská M. Fraňová S. (2018). Acute and Chronic Effects of Oral Erdosteine on Ciliary Beat Frequency, Cough Sensitivity and Airway Reactivity. Adv. Exp. Med. Biol. 1023, 1–10. 10.1007/5584_2017_48 28730380

[B72] Pappova L. Kazimierova I. Kocmalova M. (2017). Effect of inhaled and oral N-acetylcysteine on airway defense mechanism. Eur. Pharm. J. 64, 17–21. 10.1515/afpuc-2017-0002

[B73] Pavia D. Bateman J. R. Sheahan N. F. Clarke S. W. (1979). Effect of ipratropium bromide on mucociliary clearance and pulmonary function in reversible airways obstruction. Thorax 34, 501–507. 10.1136/thx.34.4.501 159510 PMC471105

[B74] Poole P. Sathananthan K. Fortescue R. (2019). Mucolytic agents versus placebo for chronic bronchitis or chronic obstructive pulmonary disease. Cochrane Database Syst. Rev. 5, CD001287. 10.1002/14651858.CD001287.pub6 31107966 PMC6527426

[B75] Prakriya M. (2009). The molecular physiology of CRAC channels. Immunol. Rev. 231, 88–98. 10.1111/j.1600-065X.2009.00820.x 19754891 PMC3253762

[B76] Rossman C. M. Lee R. M. Forrest J. B. Newhouse M. T. (1984). Nasal ciliary ultrastructure and function in patients with primary ciliary dyskinesia compared with that in normal subjects and in subjects with various respiratory diseases. Am. Rev. Respir. Dis. 129, 161–167. 10.1164/arrd.1984.129.1.161 6703474

[B77] Słoczyńska K. Kózka M. Pękala E. Marchewka A. Marona H. (2013). In vitro effect of pentoxifylline and lisofylline on deformability and aggregation of red blood cells from healthy subjects and patients with chronic venous disease. Acta Biochim. Pol. 60, 129–135. 10.18388/abp.2013_1962 23520579

[B78] Salathe M. Lipson E. J. Ivonnet P. I. Bookman R. J. (1997). Muscarinic signaling in ciliated tracheal epithelial cells: dual effects on Ca^2+^ and ciliary beating. Am. J. Physiol. 272, L301–L310. 10.1152/ajplung.1997.272.2.L301 9124382

[B79] Sanderson M. J. Dirksen E. R. (1989). Mechanosensitive and beta-adrenergic control of the ciliary beat frequency of mammalian respiratory tract cells in culture. Am. Rev. Respir. Dis. 139, 432–440. 10.1164/ajrccm/139.2.432 2536528

[B80] Schmid A. Bai G. Schmid N. Zaccolo M. Ostrowski L. E. Conner G. E. (2006). Real-time analysis of cAMP-mediated regulation of ciliary motility in single primary human airway epithelial cells. J. Cell Sci. 119, 4176–4186. 10.1242/jcs.03181 16984973

[B81] Schmid A. Sutto Z. Nlend M. C. Horvath G. Schmid N. Buck J. (2007). Soluble Adenylyl Cyclase Is Localized to Cilia and Contributes to Ciliary Beat Frequency Regulation via Production of cAMP. J. Gen. Physiol. 130, 99. 10.1085/jgp.200709784 17591988 PMC2154360

[B82] Schmid A. Meili D. Salathe M. (2014). Soluble adenylyl cyclase in health and disease. Biochim. Biophys. Acta 1842 (12 Pt B), 2584–2592. 10.1016/j.bbadis.2014.07.010 25064591 PMC4262541

[B83] Schmid A. Baumlin N. Ivonnet P. Dennis J. S. Campos M. Krick S. (2015). Roflumilast partially reverses smoke-induced mucociliary dysfunction. Respir. Res. 16, 135. 10.1186/s12931-015-0294-3 26521141 PMC4628339

[B84] Schmidt A. Salathe M. (2011). Ciliary beat co-ordination by calcium. Biol. Cell. 103, 159–169. 10.1042/BC20100120 21401526

[B85] Shah A. S. Ben-Shahar Y. Moninger T. O. Kline J. N. Welsh M. J. (2009). Motile cilia of human airway epithelia are chemosensory. Science 325, 1131–1134. 10.1126/science.1173869 19628819 PMC2894709

[B86] Shinohara K. Chen D. Nishida T. Misaki K. Yonemura S. Hamada H. (2015). Absence of Radial Spokes in Mouse Node Cilia Is Required for Rotational Movement but Confers Ultrastructural Instability as a Trade-Off. Dev. Cell. 35, 236–246. 10.1016/j.devcel.2015.10.001 26506310

[B87] Shishikura Y. Koarai A. Aizawa H. Yamaya M. Sugiura H. Watanabe M. (2016). Extracellular ATP is involved in dsRNA-induced MUC5AC production via P2Y2R in human airway epithelium. Respir. Res. 17, 121. 10.1186/s12931-016-0438-0 27677339 PMC5039824

[B88] Singh D. Abbott-Banner K. Bengtsson T. Newman K. (2018). The short-term bronchodilator effects of the dual phosphodiesterase 3 and 4 inhibitor RPL554 in COPD. Eur. Respir. J. 52, 1801074. 10.1183/13993003.01074-2018 30166326 PMC6214575

[B89] Spinelli A. M. González-Cobos J. C. Zhang X. Motiani R. K. Rowan S. Zhang W. (2012). Airway smooth muscle STIM1 and Orai1 are upregulated in asthmatic mice and mediate PDGF-activated SOCE, CRAC currents, proliferation, and migration. Pflugers Arch. 464, 481–492. 10.1007/s00424-012-1160-5 23014880 PMC3489062

[B90] Stanic P. Sonicki Z. Suchanek E. (2002). Effect of pentoxifylline on motility and membrane integrity of cryopreserved human spermatozoa. Int. J. Androl. 25, 186–190. 10.1046/j.1365-2605.2002.00348.x 12031048

[B91] Stout S. L. Wyatt T. A. Adams J. J. Sisson J. H. (2007). Nitric Oxide-dependent Cilia Regulatory Enzyme Localization in Bovine Bronchial Epithelial Cells. J. Histochem. Cytochem. 55, 433–442. 10.1369/jhc.6A7089.2007 17242464

[B92] Tamaoki J. Kondo M. Takizawa T. (1989). Effect of cAMP on cliliary function in rabbit tracheal epithelial cells. J. Appl. Physiol. 66 (3), 1035–1039. 10.1152/jappl.1989.66.3.1035 2468639

[B93] Teilmann S. C. Byskov A. G. Pedersen P. A. Wheatley D. N. Pazour G. J. Christensen S. T. (2005). Localization of transient receptor potential ion channels in primary and motile cilia of the female murine reproductive organs. Mol. Reprod. Dev. 71, 444–452. 10.1002/mrd.20312 15858826

[B94] Thomas B. Rutman A. Hirst R. A. Haldar P. Wardlaw A. J. Bankart J. (2010). Ciliary dysfunction and ultrastructural abnormalities are features of severe asthma. J. Allergy Clin. Immunol. 126 (4), 722–729. e2 10.1016/j.jaci.2010.05.046 20673980

[B95] Torphy T. J. Undem B. J. Cieslinski L. B. Luttmann M. A. Reeves M. L. Hay D. W. (1993). Identification, characterization and functional role of phosphodiesterase isozymes in human airway smooth muscle. J. Pharmacol. Exp. Ther. 265, 1213–1223. 8389856

[B96] Tucci S. M. Skalko R. G. (1978). The teratogenic effects of theophylline in mice. Toxicol. Lett. 1, 337–341. 10.1016/0378-4274(78)90017-6

[B97] Turner M. J. Matthes E. Billet A. Ferguson A. J. Thomas D. Y. Randell S. H. (2016). The dual phosphodiesterase 3 and 4 inhibitor RPL554 stimulates CFTR and ciliary beating in primary cultures of bronchial epithelia. Am. J. Physiol. Lung Cell. Mol. Physiol. 310, L59–L70. 10.1152/ajplung.00324.2015 26545902

[B98] Uz U. Chen B. Palmer J. N. Cingi C. Unlu H. Cohen N. A. (2014). Effects of Thymoquinone and Montelukast on Sinonasal Ciliary Beat Frequency. Am. J. Rhinol. Allergy 28, 122–125. 10.2500/ajra.2014.28.4010 24717949

[B99] Vasta V. Sonnenburg W. K. Yan C. Soderling S. H. Shimizu-Albergine M. Beavo J. A. (2005). Identification of a new variant of PDE1A calmodulin-stimulated cyclic nucleotide phosphodiesterase expressed in mouse sperm. Biol. Reprod. 73 (4), 598–609. 15901640 10.1095/biolreprod.104.039180

[B100] Verhey K. J. Dishinger J. Kee H. L. (2011). Kinesin motors and primary cilia. Biochem. Soc Trans. 39, 1120–1125. 10.1042/BST0391120 21936775 PMC3538878

[B101] Wanner A. Salathé M. O’Riordan T. G. (1996). Mucociliary clearance in the airways. Am. J. Respir. Crit. Care Med. 154, 1868–1902. 10.1164/ajrccm.154.6.8970383 8970383

[B102] Wanner A. (1986). Effect of ipratropium bromide on airway mucociliary function. Am. J. Med. 81, 23–27. 10.1016/0002-9343(86)90458-4 2947458

[B103] Ward C. J. Yuan D. Masyuk T. V. Wang X. Punyashthiti R. Whelan S. (2003). Cellular and subcellular localization of the ARPKD protein; fibrocystin is expressed on primary cilia. Hum. Mol. Genet. 12, 2703–2710. 10.1093/hmg/ddg274 12925574

[B104] Wodarczyk C. Rowe I. Chiaravalli M. Pema M. Qian F. Boletta A. (2009). A novel mouse model reveals that polycystin-1 deficiency in ependyma and choroid plexus results in dysfunctional cilia and hydrocephalus. PloS One 4, e7137. 10.1371/journal.pone.0007137 19774080 PMC2743994

[B105] Wohlsen A. Hirrle A. Tenor H. Marx D. Beume R. (2010). Effect of cyclic AMP-elevating agents on airway ciliary beat frequency in central and lateral airways in rat precision-cut lung slices. Eur. J. Pharmacol. 635, 177–183. 10.1016/j.ejphar.2010.03.005 20303939

[B106] Workman A. D. Cohen N. A. (2014). The effect of drugs and other compounds on the ciliary beat frequency of human respiratory epithelium. Am. J. Rhinol. Allergy 28, 454–464. 10.2500/ajra.2014.28.4092 25514481

[B107] Wright L. C. Seybold J. Robichaud A. Adcock I. M. Barnes P. J. (1998). Phosphodiesterase expression in human epithelial cells. Am. J. Physiol. 275, L694–L700. 10.1152/ajplung.1998.275.4.L694 9755101

[B108] Yaghi A. Zaman A. Cox G. Dolovich M. B. (2012). Ciliary beating is depressed in nasal cilia from chronic obstructive pulmonary disease subjects. Respir. Med. 106 (8), 1139–1147. 10.1016/j.rmed.2012.04.001 22608352

[B109] Yaghi A. Dolovich M. B. (2016). Airway Epithelial Cell Cilia and Obstructive Lung Disease. Cells 5, 40. 10.3390/cells5040040 PMC5187524 27845721

[B110] Zagoory O. Braiman A. Gheber L. Priel Z. (2001). Role of calcium and calmodulin in ciliary stimulation induced by acetylcholine. Am. J. Physiol. Cell Physiol. 280, C100–C109. 10.1152/ajpcell.2001.280.1.C100 11121381

[B111] Zagoory O. Braiman A. Priel Z. (2002). The mechanism of ciliary stimulation by acetylcholine: roles of calcium, PKA, and PKG. J. Gen. Physiol. 119, 329–339. 10.1085/jgp.20028519 11929884 PMC2311390

[B112] Zhou H. Wang X. Brighton L. Hazucha M. Jaspers I. Carson J. L. (2009). Increased nasal epithelial ciliary beat frequency associated with lifestyle tobacco smoke exposure. Inhal. Toxicol. 21, 875e81. 10.1080/08958370802555898 19555226 PMC2721908

[B113] Zhou K. Q. Green C. R. Bennet L. Gunn A. J. Davidson J. O. (2019). The Role of Connexin and Pannexin Channels in Perinatal Brain Injury and Inflammation. Front. Physiol. 10, 141. 10.3389/fphys.2019.00141 30873043 PMC6400979

[B114] Zuo H. Han B. Poppinga W. J. Ringnalda L. Kistemaker L. E. M. Halayko A. J. (2018). Cigarette smoke up-regulates PDE3 and PDE4 to decrease cAMP in airway cells. Br. J. Pharmacol. 175, 2988–3006. 10.1111/bph.14347 29722436 PMC6016635

